# Development of a novel scoring tool to predict the need for early cricothyroidotomy in trauma patients

**DOI:** 10.1016/j.sopen.2023.09.017

**Published:** 2023-09-20

**Authors:** Mary Londoño, Jeffry Nahmias, Matthew Dolich, Michael Lekawa, Allen Kong, Sebastian Schubl, Kenji Inaba, Areg Grigorian

**Affiliations:** a University of California, Irvine, Department of Surgery, Division of Trauma, Burns and Surgical Critical Care, Orange, CA, USA; b University of Southern California, Department of Surgery, Los Angeles, CA, USA

**Keywords:** Cricothyroidotomy, Trauma, Scoring tool, Severe neck injury, Gunshot wound

## Abstract

**Background:**

The lack of a widely-used tool for predicting early cricothyroidotomy in trauma patients prompted us to develop the Cricothyroidotomy After Trauma (CAT) score. We aimed to predict the need for cricothyroidotomy within one hour of trauma patient arrival.

**Methods:**

Derivation and validation datasets were obtained from the Trauma Quality Improvement Program (TQIP) database. Logistic modeling identified predictors, and weighted averages were used to create the CAT score. The score's performance was assessed using AUROC.

**Results:**

Among 1,373,823 derivation patients, <1 % (*n* = 339) underwent cricothyroidotomy within one hour. The CAT score, comprising nine predictors, achieved an AUROC of 0.88. Severe neck injury and gunshot wound were the strongest predictors. Cricothyroidotomy rates increased from 0.4 % to 9.3 % at scores of 5 and 8, respectively. In the validation set, the CAT tool yielded an AUROC of 0.9.

**Conclusion:**

The CAT score is a validated tool for predicting the need for early cricothyroidotomy in trauma patients. Further research is necessary to enhance its utility and assess its value in trauma care.

## Highlights

• No widely-used tool exists to predict early cricothyroidotomy in trauma patients
• Predicting cricothyroidotomy can prevent adverse hypoxemic events
• Cricothyroidotomy After Trauma (CAT) scoring tool predicts cricothyroidotomy need
• Severe neck injury and gunshot wound are the strongest cricothyroidotomy predictors
• The CAT tool can help trauma centers prepare for immediate cricothyroidotomy

## Introduction

The first documented surgical airway occurred in 1546 [1]. Over 300 years later, in the early 1900s, cricothyroidotomy emerged as a formal surgical technique popularized by Dr. Chevalier Jackson from Philadelphia. However, he soon abandoned the procedure citing a high rate of tracheal stenosis [2]. The procedure returned to mainstream practice in the 1970s when Drs. Brantigan and Grow published their experience with a low complication rate [3]. Cricothyroidotomy is now the emergent procedure of choice for adult patients with a failed airway [[4], [5], [6]]. Trauma is by far the most common indication for an emergent cricothyroidotomy but it still only occurs in <1 % of trauma patients [[7], [8], [9], [10]].

Emergent cricothyroidotomy is recommended when other methods of securing the airway have been exhausted or in a cannot-intubate-cannot‑oxygenate (CICO) scenario; however this point of transition remains poorly defined and variable among providers [4,5,11]. Even surgeons may hesitate to perform cricothyroidotomies due to anxiety, decision-making delay, and ill preparation– both cognitively and physically, in terms of the availability of appropriate equipment and supplies [4,6,12]. Guidelines from the Difficult Airway Society, Eastern Association for the Surgery of Trauma, and American College of Emergency Physicians suggest that up to three failed endotracheal intubations can be attempted prior to cricothyroidotomy [1,4,[11], [12], [13]]. However, this may not always be appropriate and uniformly applicable to all trauma patients as morbidity, including hemodynamic/hypoxic adverse events, can occur even after just one failed intubation attempt and may negatively impact patient outcomes (e.g., patients with a traumatic brain injury) [[14], [15], [16]]. Unfortunately, many cricothyroidotomies are delayed until critical hypoxemia has already occurred [6]. These delays have been associated with significant adverse outcomes, ranging from airway trauma and hypoxia to anoxic brain injury, cardiopulmonary arrest, and death [4,11,12].

Given the grave consequences of uncertainty and lack of preparation for cricothyroidotomies, the ability to predict which trauma patients may require cricothyroidotomy soon after arrival might be helpful [11]. In addition, prognostication of which trauma patients are at risk to require emergent cricothyroidotomy may help guide future research and compare quality outcomes between centers [5,6]. Therefore, this study aimed to identify and stratify risk factors and develop a novel Cricothyroidotomy After Trauma (CAT) score to predict the need for cricothyroidotomy within one-hour of arrival for trauma patients.

## Methods

This study was deemed exempt by our Institutional Review Board as it utilizes a national deidentified database. We performed a retrospective analysis using the 2017–2019 Trauma Quality Improvement Program (TQIP) database, which is a conglomerate of over 875 participating trauma centers across the United States [17]. The TQIP database was queried for patients ≥18 years old. We excluded all patients that were transferred from another hospital. Patients were divided into two sets: a derivation (using 2017–2018 data) and validation set (using 2019 data). The primary outcome was emergent cricothyroidotomy performed within one-hour of arrival. This was defined using International Classification of Diseases version 10 (ICD-10) procedure code *0B110F4*. After discussion among coauthors and review of the literature, we identified variables available in TQIP that may be considered independent predictors of requiring an emergent cricothyroidotomy [[6], [7], [8],^12^,^18^,^19^]. We then performed a univariable logistic regression analysis to determine which of these variables were associated with significant risk of emergent cricothyroidotomy defined by a *p*-value <0.2. The variables that were ultimately selected included male sex, penetrating trauma, severe injury to the head, neck or face, comorbid cerebrovascular accident, comorbid mental/personality disorder, systolic blood pressure < 90 mmHg, and tachycardia >120 beats per minute.

A three-step methodology was used to then develop the CAT score. First, by comparing patients who underwent cricothyroidotomy (cric^*+*^) to patients who did not undergo cricothyroidotomy (cric^*−*^), we ran a multiple logistic regression model using the aforementioned variables to determine the independent risk of emergent cricothyroidotomy. We considered independent predictors to have a *p*-value <0.05. Next, the weighted and relative impact of each covariate was used to derive an integer value for that predictor. We did this using a validated approach to simplify the scoring tool [[20], [21], [22], [23]]. Each of the variables were multiplied by a factor so that the smallest odds ratio was transformed to a value of 1 and the strongest predictor assigned a CAT score value of 3. We then confirmed the accuracy of our scoring tool using the area under the receiver operating curve (AUROC). The same three-step methodology was then applied to the 2019 validation set to confirm that we were able to achieve a similar AUROC using a completely different group of patients. And finally, we then used the CAT tool to identify the rate of cricothyroidotomy within one-hour of arrival for various scores.

We collected basic demographics such as age, sex and comorbidities, including hypertension, diabetes, congestive heart failure, cerebrovascular accident, myocardial infarction, bleeding disorder, anticoagulant therapy, chronic obstructive pulmonary disease, cirrhosis, chronic renal failure, dementia, mental/personality disorder (defined by presence of pre-injury depressive disorder, bipolar disorder, schizophrenia, borderline or antisocial personality disorder, and/or adjustment disorder/post-traumatic stress disorder), substance abuse, alcoholism, and current smoking. Cerebrovascular accident is defined in TQIP by the following: A history prior to injury of a cerebrovascular accident (embolic, thrombotic, or hemorrhagic) with persistent residual motor sensory or cognitive dysfunction (e.g., hemiplegia, hemiparesis, aphasia, sensory deficit, impaired memory). Vitals on arrival were recorded categorically and included hypotension (systolic blood pressure < 90 mmHg), tachypnea (respiratory rate > 22 breaths per minute), and tachycardia (heart rate > 120 beats per minute). Injury characteristics included injury severity score (ISS), mechanism of injury, and specific injury or injuries according to ICD-10 diagnosis codes available in the TQIP database. Severe injury was defined by an abbreviated injury scale (AIS) ≥3. Additional outcomes collected included mortality, total hospital length of hospital stay (LOS) in days, intensive care unit (ICU) LOS in days, ventilator days, in-hospital complications, and discharge disposition. Categorical variables were represented as totals with percentages and compared with chi-square testing. Continuous variables were reported as medians with interquartile range and analyzed with a Mann-Whitney *U* test. All *p*-values were double sided with a statistical significance level of <0.05. All analyses were performed with IBP SPSS Statistics for Windows (version 28, IBM Corp, Armonk, NY).

## Results

### Demographics of Cric+ and Cric− patients

From 1,373,823 patients in the derivation set, 339 (<1 %) underwent emergent cricothyroidotomy. Compared to the cric− group, patients in the cric^+^ group were significantly younger (median age, 40 vs 55-years, *p* < 0.001), and had higher rates of males (87.3 % vs 58.5 %, *p* *<* *0.001*), hypotension (23.2 % vs 3.6 %, *p* < 0.001), tachycardia (27.5 % vs 7.0 %, *p* < 0.001), and tachypnea on admission (34.7 % vs 16.0 %, *p* < 0.001). The cric^+^ group also had a higher rate of comorbid mental/personality disorder (17.1 % vs 10.4 %, *p* < 0.001), alcoholism (8.3 % vs 5.5 %, *p* = 0.028), and substance abuse (10.3 % vs 6.6 %, *p* = 0.005) (Table 1).

**Table 1:** Demographics of trauma patients in the derivation set who did not undergo early cricothyroidotomy vs those who did undergo early cricothyroidotomy.

Characteristic	Cric^−^	Cric^+^	p-value
	(*n* = 1,373,484)	(n = 339)	
Age, years, median (IQR)	55 (33–72)	40 (28–52)	<0.001
Male, n (%)	803,794 (58.5 %)	296 (87.3 %)	<0.001
ISS, median (IQR)	10 (4–10)	19 (9–27)	<0.001
Vitals on admission, n (%)			
Hypotensive (SBP <90 mmHg)	48,666 (3.6 %)	75 (23.2 %)	<0.001
Tachypneic (>22/min)	213,086 (16.0 %)	103 (34.7 %)	<0.001
Tachycardic (>120/min)	93,468 (7.0 %)	91 (27.5 %)	<0.001
Alcohol positive, n (%)	190,960 (29.7 %)	69 (30.4 %)	0.824
Drug screen positive, n (%)	176,423 (44.2 %)	68 (46.3 %)	0.609
Comorbidities, n (%)			
Alcoholism	76,012 (5.5 %)	28 (8.3 %)	0.028
Anticoagulant therapy	129,147 (9.4 %)	5 (1.5 %)	<0.001
Bleeding disorder	18,843 (1.4 %)	0 (0.0 %)	0.030
Cerebrovascular accident	39,285 (2.9 %)	5 (1.5 %)	0.126
Chronic renal failure	24,291 (1.8 %)	1 (0.3 %)	0.040
Cirrhosis	13,204 (1.0 %)	1 (0.3 %)	0.209
Congestive heart failure	59,345 (4.3 %)	2 (0.6 %)	<0.001
COPD	93,052 (6.8 %)	11 (3.2 %)	0.010
Current smoker	265,006 (19.3 %)	54 (15.9 %)	0.116
Dementia	83,530 (6.1 %)	1 (0.3 %)	<0.001
Diabetes	189,687 (13.8 %)	22 (6.5 %)	<0.001
Hypertension	484,969 (35.3 %)	49 (14.5 %)	<0.001
Mental/personality disorder	142,179 (10.4 %)	58 (17.1 %)	<0.001
Myocardial infarction	12,352 (0.9 %)	2 (0.6 %)	0.546
Substance abuse	90,206 (6.6 %)	35 (10.3 %)	0.005

Cric^−^ = patients who did not undergo cricothyroidotomy; Cric^+^ = patients who underwent cricothyroidotomy; ISS = Injury Severity Score; IQR = interquartile range; SBP = systolic blood pressure; COPD = Chronic Obstructive Pulmonary Disease.

### Injury characteristics of Cric+ and Cric− patients

The cric+ group had a higher median ISS (19 vs 10, *p* < 0.001), and more commonly presented after a gunshot (35.7 % vs 4.7 %, *p* < 0.001) or stab wound mechanism (28.6 % vs 4.5 %, *p* < 0.001). Cric^+^ patients suffered more injuries to the head and neck regions compared to cric^−^ patients, including fracture of skull or face (42.2 % vs 13.2 %, *p* < 0.001), cervical fracture (18.3 % vs 4.8 %, *p* < 0.001), traumatic brain injury (26.3 % vs 15.5 %, *p* < 0.001), spine fracture (24.8 % vs 15.4 %, *p* < 0.001), and spinal cord injury (3.2 % vs 1.5 %, *p* = 0.009) (Table 2).

**Table 2:** Characteristics of mechanisms and injuries for trauma patients in the derivation set who did not undergo early cricothyroidotomy vs those who did undergo early cricothyroidotomy.

Injury	Cric^−^	Cric^+^	p-value
	(n = 1,373,484)	(n = 339)	
Blunt mechanism, n (%)			
Fall	659,669 (48.0 %)	17 (5.0 %)	<0.001
Pedestrian	53,622 (3.9 %)	8 (2.4 %)	0.142
Bicycle	30,857 (2.2 %)	2 (0.6 %)	0.040
Motorcycle	74,928 (5.5 %)	13 (3.8 %)	0.189
Motor vehicle collision	291,356 (21.2 %)	54 (15.9 %)	0.017
Penetrating mechanism, n (%)			
Gunshot	64,776 (4.7 %)	121 (35.7 %)	<0.001
Stab	61,277 (4.5 %)	97 (28.6 %)	<0.001
Injuries, n (%)			
Traumatic brain injury	212,136 (15.5 %)	89 (26.3 %)	<0.001
Fracture of skull or face	181,408 (13.2 %)	143 (42.2 %)	<0.001
Cervical fracture	65,888 (4.8 %)	62 (18.3 %)	<0.001
Cervical cord	13,776 (1.0 %)	10 (2.9 %)	<0.001
Spine fracture	211,616 (15.4 %)	84 (24.8 %)	<0.001
Spinal cord	20,703 (1.5 %)	11 (3.2 %)	0.009
Upper extremity fracture	177,672 (12.9 %)	31 (9.1 %)	0.038
Lung	159,217 (11.6 %)	96 (28.3 %)	<0.001
Pneumothorax	80,900 (5.9 %)	52 (15.3 %)	<0.001
Hemothorax	22,346 (1.6 %)	15 (4.4 %)	<0.001
Hemopneumothorax	27,343 (2.0 %)	20 (5.9 %)	<0.001
Heart	7581 (0.6 %)	6 (1.8 %)	0.002
Diaphragm	6954 (0.5 %)	6 (1.8 %)	0.001
Kidney	15,999 (1.2 %)	4 (1.2 %)	0.979
Small intestine	12,179 (0.9 %)	13 (3.8 %)	<0.001
Spleen	30,270 (2.2 %)	11 (3.2 %)	0.192
Liver	32,367 (2.4 %)	17 (5.0 %)	0.001
Colon	10,750 (0.8 %)	10 (2.9 %)	<0.001
Rectum	1408 (0.1 %)	1 (0.3 %)	0.268
Pelvic fracture	90,082 (6.6 %)	17 (5.0 %)	0.251
Lower Extremity fracture	381,901 (27.8 %)	25 (7.4 %)	<0.001

Cric− = patients who did not undergo cricothyroidotomy; Cric+ = patients who underwent cricothyroidotomy.

### Outcomes of the derivation set for Cric+ and Cric− patients

The median hospital LOS (15 days vs 4 days, *p* < 0.001) and ICU LOS (5 days vs 3 days, *p* < 0.001) differed significantly between cric^+.^and cric^−^ patients. The occurrence of several in-hospital cardiovascular and pulmonary complications was higher in the cric^+^ group compared to the cric− group, including cardiac arrest (8.3 % vs 0.7 %, *p* < 0.001), acute respiratory distress syndrome (2.4 % vs 0.3 %, *p* < 0.001), and pulmonary embolism (1.5 % vs 0.3 %, *p* < 0.001) (see Table 3). Those in the cric^+^ group also had increased mortality compared to cric− patients (28.9 % vs 4.1 %, *p* < 0.001) (Table 3).

**Table 3:** Outcomes of trauma patients in the derivation set who did not undergo early cricothyroidotomy vs those who did undergo early cricothyroidotomy.

Outcome	Cric^−^	Cric^+^	p-value
	(n = 1,373,484)	(n = 339)	
LOS, days, median (IQR)	4 (2–6)	15 (10–24)	<0.001
ICU LOS, days, median (IQR)	3 (2–6)	5 (3−10)	<0.001
Ventilator, days, median (IQR)	3 (1–7)	3 (2–8.25)	0.190
Hospital complication, n (%)			
Stroke	3015 (0.2 %)	4 (1.2 %)	<0.001
Cardiac arrest	9860 (0.7 %)	28 (8.3 %)	<0.001
Myocardial infarction	2117 (0.2 %)	0 (0.0 %)	0.469
CLABSI	501 (0.0 %)	1 (0.3 %)	0.013
ARDS	3600 (0.3 %)	8 (2.4 %)	<0.001
Ventilator associated pneumonia	5614 (0.4 %)	10 (2.9 %)	<0.001
Pulmonary embolism	3753 (0.3 %)	5 (1.5 %)	<0.001
Deep vein thrombosis	6995 (0.5 %)	13 (3.8 %)	<0.001
Acute kidney injury	6342 (0.5 %)	5 (1.5 %)	0.006
Deep SSI	1309 (0.1 %)	2 (0.6 %)	0.003
Superficial SSI	1129 (0.1 %)	7 (2.1 %)	<0.001
Sepsis	3596 (0.3 %)	3 (0.9 %)	0.025
Unplanned intubation	11,255 (0.8 %)	1 (0.3 %)	0.284
Unplanned return to OR	5482 (0.4 %)	12 (3.5 %)	<0.001
Discharge disposition, n (%)			<0.001
Home	705,215 (60.5 %)	122 (40.8 %)	
Inpatient rehabilitation	25,752 (1.9 %)	25 (7.4 %)	
Intermediate or long-term care	142,139 (10.3 %)	27 (8.0 %)	
Skilled nursing facility	201,767 (17.3 %)	13 (4.3 %)	
Mortality, n (%)	55,961 (4.1 %)	98 (28.9 %)	<0.001

Cric− = patients who did not undergo cricothyroidotomy; Cric+ = patients who underwent cricothyroidotomy; LOS = Length of Stay; IQR = interquartile range; ICU = Intensive Care Unit; CLABSI = Central Line Associated Bloodstream Infection; ARDS = Acute Respiratory Distress Syndrome; SSI = Surgical Site infection; OR = Operating Room.

### Results of CAT scoring tool development

**Fig. 1:**
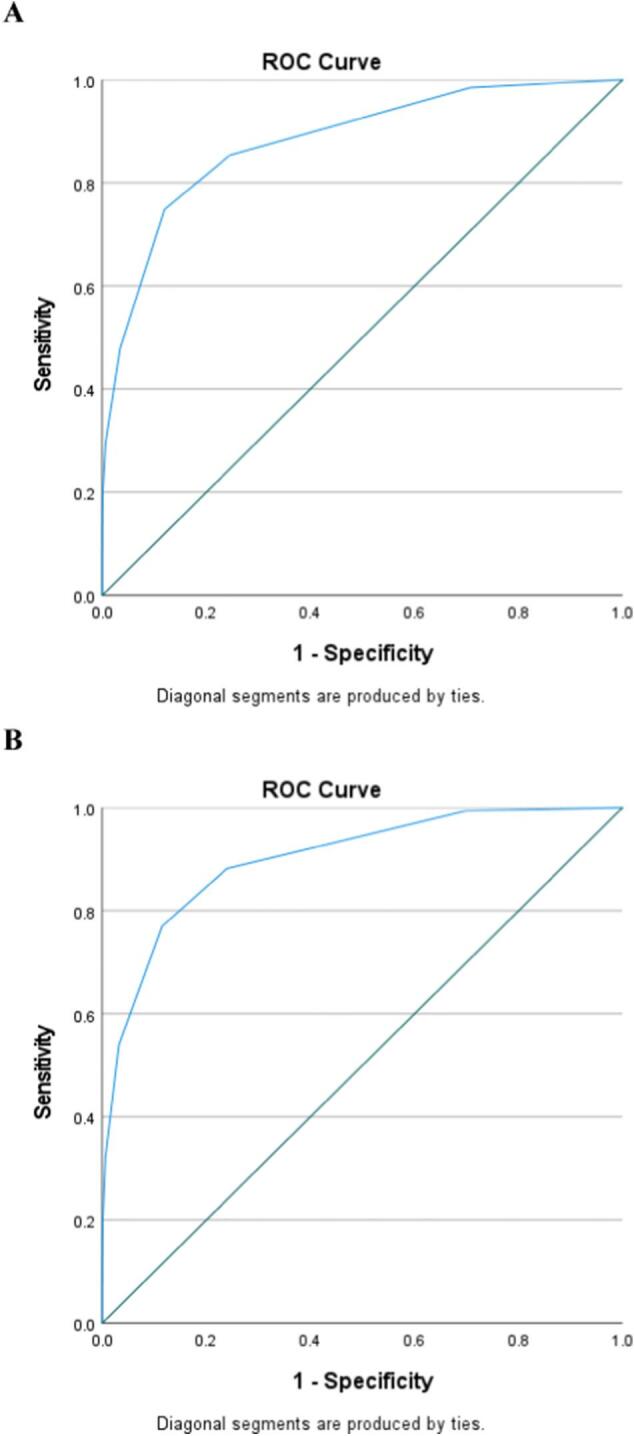
Area under the curve for development of the Cricothyroidotomy After Trauma score **A**. Derivation set [AROC = 0.88] **B**. Validation set [AROC = 0.90].

**Fig. 2:**
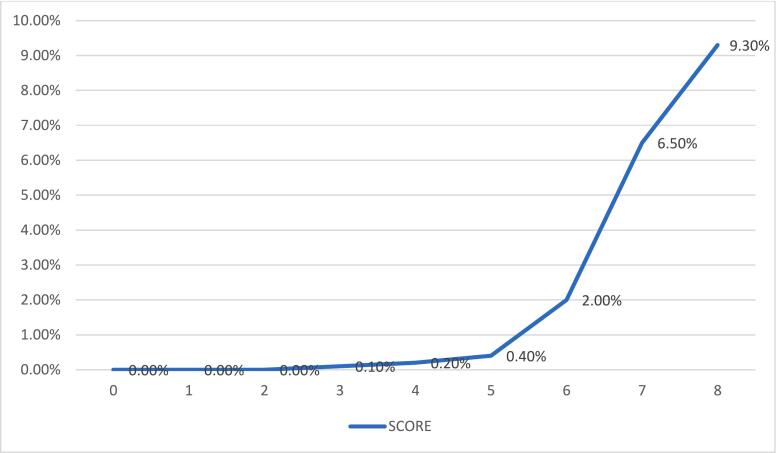
Early cricothyroidotomy rate for various Cricothyroidotomy After Trauma scores.

The strongest predictor of emergent cricothyroidotomy was found to be severe neck injury (AIS > 3) (OR 35.16, CI 19.92–62.08, *p* < 0.001), followed by penetrating trauma (OR 6.70, CI 3.24–13.84, *p* < 0.001) (Table 4). The AROC for the CAT scoring tool was 0.88 (CI 0.86–0.90) (Fig. 1A). In the validation set, 743,036 patients had an emergent cricothyroidotomy rate of <1 %. The AROC for the validation set was 0.90. Applying the tool, the emergent cricothyroidotomy rate increased steadily from 0.4 % to 2.0 % to 6.5 %, then 9.3 % at scores of 5, 6, 7, and 8, respectively (Fig. 2).

**Table 4:** Development of the cricothyroidotomy after trauma scoring tool.

Variable	Points
Demographics	
Male	1
Comorbidities	
Mental/personality disorder^a^	1
Cerebrovascular accident	1
Penetrating trauma	2
Vitals on admission	
Systolic blood pressure < 90 mmHg	1
Tachycardic (>120/min)	1
Severe injury^b^	
Head	1
Face	2
Neck	3
Maximum score	13
ROC	0.880
95 % CI for ROC	0.860–0.901

_a_ Defined by presence of pre-injury depressive disorder, bipolar disorder, schizophrenia, borderline or antisocial personality disorder, and/or adjustment disorder/post-traumatic stress disorder.

b Defined by abbreviated injury scale ≥3.

## Discussion

There is currently no well-established tool for predicting the need for emergent cricothyroidotomy for trauma patients. This large, national database study identified predictive risk factors for cricothyroidotomy including male sex, severe head, neck or face injury, penetrating mechanism of injury, comorbid cerebrovascular accident or mental/personality disorder, and vitals on arrival. In addition, a novel CAT score was then developed and validated to predict the need for emergent cricothyroidotomy in trauma patients, which can be calculated soon after arrival.

The trauma patient in the CICO situation is immediately vulnerable to life-threatening and devastating hypoxic, cardiopulmonary, and anoxic brain injuries. The decision to perform a cricothyroidotomy to provide oxygenation and ventilation to this type of patient is one that cannot and should not be delayed for any reason. Thus, the ability to predict which patients require cricothyroidotomy shortly after arrival may help improve survival in these relatively rare trauma patients [5,6,8,11,12,24,25]. Many non-surgeons may hesitate to perform emergent cricothyroidotomy for a variety of reasons, thereby potentially increasing airway trauma and morbidity from intubation attempts and increasing the risk of adverse outcomes caused by hypoxia [4,6,11,12,15,16,25]. Previous attempts at developing a similar scoring tool have been limited by the focus on predicting difficult airways/intubation, but not specifically the need for cricothyroidotomy, and have been limited to the emergency department setting (emergency physicians, residents, etc.) exclusively [26]. A more recent scoring tool from Japan, developed by Okada et al., also sought to predict emergency front of neck airway access, but studied both cricothyroidotomy and emergency needle cannula and lacked external generalizability [8]. Definitive airway management by pre-hospital emergency medical services was included in their scoring tool, a practice which is generally discouraged and remains controversial in much of the United States [27,28]. Furthermore, they did not find penetrating trauma to be a significant predictor of emergency airway access, although this may be because Japan, overall, has a much lower incidence of penetrating violence, compared to the United States [29,30]. Furthermore, because vitals on admission are the only quantitatively measurable variables required for the CAT score, the tool can be used quickly and accurately in the trauma bay soon after patient arrival and may help to alleviate the anxiety, confusion, and lack of preparation often associated with the decision to perform cricothyroidotomies [[4], [5], [6],^25^,^26^].

The association between severe neck trauma and emergent cricothyroidotomy has previously been demonstrated [7,8,12,19]. Additionally, facial and head trauma also significantly increases the risk for surgical airway, with one study citing that over 30 % of patients who received a cricothyroidotomy also sustained facial injuries [7,19]. Male sex was more predictive of cricothyroidotomy than female sex, likely explained by widely-reported statistics that males have higher traumatic injury incidence, ISS, and higher likelihood of ICU admission than their female counterparts [[31], [32], [33]]. Overall hospital and ICU LOS was higher in the cric^+^ group – an unsurprising finding given their higher ISS and mechanisms of injury. Likewise, the findings of higher rates of hospital complications and overall mortality are to be expected given the extent of injury present among trauma patients in CICO situations [[6], [7], [8]].

Patient comorbidities have not been included in previous cricothyroidotomy scoring tools; however, in our CAT score, two specific comorbidities were found to be independent predictors for cricothyroidotomy: mental/personality disorder and cerebrovascular accident. Both of these conditions have a neurologic component and thus may prevent these patients from participating with clinical treatment and put them at increased risk for self-inflicted penetrating injuries to the head/face [34]. Additionally, limited studies have evaluated the outcomes of trauma patients with mental/personality disorder, suggesting that hospital complications and mortality may be higher than the baseline population [[34], [35], [36]]. Finally, in regard to cerebrovascular accidents, there is a known correlation with cervical injuries and hemodynamic instability that may put these patients at increased risk of requiring a cricothyroidotomy [37,38] Regardless, providers should be aware of these risk factors, especially when having difficulty with an initial attempt at intubation and/or in the setting of CICO.

There are several inherent limitations to this study. Given the use of a retrospective national database, our data is subject to misclassification and missing variables. It is also likely there are other variables either not accounted for in the study or not included in the database that may predict the need for cricothyroidotomy, including number of failed intubation attempts, reason for failed intubation, Mallampati score, and body habitus [7,8,18,19]. Comorbidities may not be known at the time of patient presentation unless emergency medical personnel was able to provide this information. Similarly, the extent of all the injuries may not be readily known at the time of presentation. Additionally, by using the TQIP database, our data lacks a level of granularity with respect to injuries and anatomy often relevant to cricothyroidotomy performance. Specific cervical and laryngeal anatomic abnormalities of the patient, findings on laryngoscopy, presence of inhalational injuries, upper versus lower airway injuries, equipment used, and presence of blood or vomitus in the airway are all details not accounted for that can affect the need for cricothyroidotomies [19,26,39,40]. Likewise, because the dataset only records presence or absence of hospital complications and not when the complication occurred, we are unable to determine the temporal association of complication and cricothyroidotomy. Finally, a major limitation is that the current CAT tool can only predict at most a nearly 10 % chance of requiring a cricothyroidotomy. However, this is an exponentially increased risk compared to the general trauma patient and we believe this CAT tool is a starting point that can be refined with future prospective studies that include some of the aforementioned variables that are not contained within TQIP.

## Conclusion

The CAT score is a novel and validated scoring tool that was developed to predict the need for emergent cricothyroidotomy in adult trauma patients. The CAT score is distinguished from other existing scoring tools by its applicability to all adult trauma patients and inclusion of comorbidities. We have demonstrated and validated that the likelihood of cricothyroidotomy increases as a function of the CAT score. This study may help trauma centers develop protocols to determine the setting in which immediate cricothyroidotomy is warranted, thereby preventing delays that lead to critical hypoxemia and eliminating adverse effects of futile repeat failed intubation attempts. This may include reserving monitored beds (i.e., telemetry level of care), respiratory therapists, ventilators, etc. for higher risk patients. Additionally, the CAT score can be used to stratify patients in future studies examining outcomes related to patients requiring a surgical airway. Future prospective research is needed to evaluate the efficacy of the CAT score and improve upon this existing framework with prospectively collected variables that are not contained within TQIP.

## Funding source

This research did not receive any specific grant from funding agencies in the public, commercial, or not-for-profit sectors.

## Ethical approval

This research used a deidentified national database and so a waiver was issued from our local institutional review board.

## CRediT authorship contribution statement

All authors contributed to the study conception and design. Data collection and statistics were performed by AG. All authors participated in data analysis. The first draft of the manuscript was written by ML and all authors commented on previous versions of the manuscript. All authors read and approved the final manuscript.

## Declaration of competing interest

The authors report no conflicts of interest.
